# Diagnostic Accuracy of Robot-Guided, Software Based Transperineal MRI/TRUS Fusion Biopsy of the Prostate in a High Risk Population of Previously Biopsy Negative Men

**DOI:** 10.1155/2016/2384894

**Published:** 2016-11-21

**Authors:** Malte Kroenig, Kathrin Schaal, Matthias Benndorf, Martin Soschynski, Philipp Lenz, Tobias Krauss, Vanessa Drendel, Gian Kayser, Philipp Kurz, Martin Werner, Ulrich Wetterauer, Wolfgang Schultze-Seemann, Mathias Langer, Cordula A. Jilg

**Affiliations:** ^1^Department of Urology, Medical Center, Faculty of Medicine, University of Freiburg, Hugstetter Strasse 55, 79106 Freiburg, Germany; ^2^Department of Radiology, Medical Center, Faculty of Medicine, University of Freiburg, Hugstetter Strasse 55, 79106 Freiburg, Germany; ^3^Department of Clinical Pathology, Medical Center, Faculty of Medicine, University of Freiburg, Breisacher Strasse 115a, 79106 Freiburg, Germany

## Abstract

*Objective*. In this study, we compared prostate cancer detection rates between MRI-TRUS fusion targeted and systematic biopsies using a robot-guided, software based transperineal approach.* Methods and Patients*. 52 patients received a MRIT/TRUS fusion followed by a systematic volume adapted biopsy using the same robot-guided transperineal approach. The primary outcome was the detection rate of clinically significant disease (Gleason grade ≥ 4). Secondary outcomes were detection rate of all cancers, sampling efficiency and utility, and serious adverse event rate. Patients received no antibiotic prophylaxis.* Results*. From 52 patients, 519 targeted biopsies from 135 lesions and 1561 random biopsies were generated (total *n* = 2080). Overall detection rate of clinically significant PCa was 44.2% (23/52) and 50.0% (26/52) for target and random biopsy, respectively. Sampling efficiency as the median number of cores needed to detect clinically significant prostate cancer was 9 for target (IQR: 6–14.0) and 32 (IQR: 24–32) for random biopsy. The utility as the number of additionally detected clinically significant PCa cases by either strategy was 0% (0/52) for target and 3.9% (2/52) for random biopsy.* Conclusions*. MRI/TRUS fusion based target biopsy did not show an advantage in the overall detection rate of clinically significant prostate cancer.

## 1. Introduction

Prostate cancer (PCa) is the second most common cancer and third most leading cause of death of men in the western world [1]. Correct risk stratification and early detection of PCa are crucial for optimal treatment of high risk patients and to avoid overtreatment in low risk patients. Risk stratification is based on histologic analysis of invasive prostate biopsies, which are indicated by elevated prostate specific antigen (PSA) levels. Low specificity (6–66%) [2] of PSA tests and low detection rates (27%–43%) [3] of transrectal ultrasound (TRUS) guided 12-core biopsies hamper detection rates and cause unwanted side effects such as infections.

Introducing a transperineal approach for biopsy, significantly lowered complication rates, however, required general anesthesia instead [4, 5]. Detection rates were reported comparable to the transrectal approach [6, 7]. The introduction of multiparametric magnetic resonance imaging (mpMRI) for planning biopsies further increased biopsy efficiency [8, 9]. Particularly, MRI/TRUS fusion biopsy improved detection rates of clinically significant prostate cancer with fewer biopsies necessary as reported by several reviews [8, 10, 11]. Individual studies, however, showed heterogeneous results. Several studies report no clear advantage of men mpMRI/TRUS-guided targeted biopsy and recommend the combination of targeted and systematic biopsies for the detection of prostate cancer [12].

The recently released platform (iSR'obot Mona Lisa) by Biobot surgical LTD combines several technical aspects to further refine the mpMRI/TRUS fusion approach. The system uses an “elastic” fusion algorithm to account for deformations from transrectal TRUS imaging compared to undeformed MRI imaging. It also uses a robotic arm, which allows all biopsies to be taken from the same two perineal 1 mm incisions and also defines the penetration depth automatically. This allows for a short procedure time and user friendly handling. In our study, we employed this system to evaluate the detection rate of clinically significant disease (Gleason grade ≥ 4) as primary outcome detection rate of all cancers, sampling efficiency and utility, and serious adverse event rate as secondary outcomes. Transperineal targeted biopsies were compared to volume adapted transperineal systematic random biopsies according to the Ginsburg study scheme [13].

## 2. Patients and Methods

### 2.1. Patients

Between 10/2015 and 05/2016, 52 patients (median age of 66, interquartile range (IQR): 60–71.75) with elevated PSA (median 8.75 ng/ml, IQR: 5.86–13.04) or clinical suspicion for PCa and prior negative 12-core transrectal ultrasound guided biopsy received MRI/TRUS fusion biopsy of the prostate in our institution. For our retrospective analysis, primary outcome was the detection rate of clinically significant disease (Gleason grade ≥ 4). Secondary outcomes were detection rate of all cancers, sampling efficiency and utility, and serious adverse event rate. Patients received no antibiotic prophylaxis.

### 2.2. Multiparametric Magnetic Resonance Imaging (mpMRI)

Each patient received a multiparametric MRI of the prostate prior to MRI/TRUS fusion biopsy using surface coils only. In each patient, triplanar T2-weighted turbo spin-echo MR imaging, axial unenhanced T1-weighted MR imaging, and diffusion weighted imaging sequences with ADC (apparent diffusion coefficient) maps were acquired. Contrast agent was applied when evaluated as necessary based on the current PI-RADS version 2 lexicon. The current PI-RADSv2 lexicon describes lesion in the transitional zone primarily on T2 weighted images and refers to DWI in cases of PI-RADS 3 to decide whether to upgrade to PI-RADS 4. Contrast agent is not a feature to describe (or detect) transitional zone lesions. In the peripheral zone, the lexicon primarily relies on DWI. Contrast agent only is administered in cases of PI-RADS 3 (i.e., moderately low ADC value with no hyperintense signal in the corresponding high b-value image).

Contrast agent was applied when a PI-RADS 3 lesion was observed in the peripheral zone. A board-certified radiologist decided about contrast agent administration. Our study reports on the diagnostic accuracy of PI-RADSv2. We therefore suppose that the applied decision scheme to administer i.v. contrast reflects the PI-RADSv2 diagnostic accuracy.

Lesions were classified by a board-certified radiologist according to the PI-RADS version 2 lexicon into categories 2 to 5 (category 1 denoting “no tumor”). For a workflow description of the diagnostic process, refer to [14]. Before the MRI/TRUS fusion biopsy, lesions were segmented manually in the Biobot software environment by a radiologist trained in prostate MRI evaluation.

### 2.3. iSR'obot MonaLisa (Biobot)

The MonaLisa system uses a software controlled robotic arm, which is mounted to the operation table. The system utilizes two software components: UroFusion for preparation of the mpMRI images and the registration of suspected cancer lesions. UroBiopsy is used to generate the TRUS based model, fuse mpMRI, and TRUS model and perform the biopsy.

T2 weighted mpMRI images were uploaded into UroFusion. A team of experienced radiologists (Matthias Benndorf, Philipp Lenz, Tobias Krauss, Mathias Langer) manually contoured the prostate and a 3D model was generated. Tumor volumes were contoured within the T2 weighted images only but were defined using T2, DWI, and DCE-MRI from the original mpMRI dataset according to the recommendation of PiRADS version 2 classification.

When the patient was under general anesthesia, he was put in lithotomy position. Antiseptic washing was performed. No local anesthesia or antibiotic prophylaxis was given.

The MonaLisa system was connected to a BK3000 ultrasound machine with a transrectal probe. The transrectal probe was mounted onto the robotic arm of the system and covered by a rigid plastic sheath to allow the probe to be automatically moved tension-free within the sheath. The sheath was prefilled with Instillagel® to avoid air bubbles and allow for optimal contact of the probe with the sheath and consequently with the tissue. The sheath was then inserted into the rectum in a position to allow for optimal coverage of the prostate within a 133 mm scanning range and optimal image quality. When start and end points for the scanning process were defined within the UroBiopsy software, transversal images were recorded every 0.5 mm. From this dataset, a 3D model was generated. The mpMRI 3D model was uploaded into the software and coregistered to the ultrasound model. To account for prostate deformation due to the transrectal ultrasound probe, the software calculates an algorithm, which fits the mpMRI model onto the TRUS model. The same algorithm is then applied to the cancer lesions. The final model shows the TRUS based outline of the prostate and the defined tumor volumes from the mpMRI. Target and random biopsies were then planned. We used the volume adapted Ginsburg study group scheme for planning the random biopsies [13]. All biopsies were taken from only two 1 mm incisions. The robot arm contains a sterile needle guide where the biopsy needle was inserted. The whole robotic arm was covered with a sterile cover to prevent contamination. A software controlled stop bar defines the penetration depth individually for each biopsy position. We used a reusable biopsy gun (Uromed REF6020) with trocar shaped biopsy needles (Uromed REF 6025.10). Biopsy cores were collected in formalin. When the robot arm had defined the penetration angle and the penetration depth, the needle was inserted and the biopsy gun was released. Incisions were covered with a small plaster. Every patient received a transurethral catheter overnight.

### 2.4. Histopathologic Analysis

Each formalin-fixed biopsy was macroscopically measured in 2 dimensions and put in a tissue cassette separately for further tissue processing and paraffin-embedding. Eight H&E stained tissue sections of each paraffin block were histologically evaluated by a urological pathologist. For each tumor, positive biopsy percentage of tumor, tumor type (almost exclusively “conventional” acinar adenocarcinoma), and Gleason Patterns integrated in ISUP-Grade Groups [15] were documented in a standard histopathologic report as well as histologic description of the other tumor negative biopsies. In case of suspect findings, immunohistochemistry was performed to rule out or confirm invasive PC or to determine special cancer subtypes/differentiation.

### 2.5. Statistical Analysis

Descriptive statistics were done by calculating mean ± standard deviation (SD), median and interquartile range (IQR), and *p* value using SPSS© software (SPSS statistics 22, IBM).

## 3. Results

52 patients (median age 66 years, interquartile range (IQR): 60–71.75) with elevated PSA (median 8.75 ng/ml, IQR: 5.86–13.04) or clinical suspicion for PCa and prior negative 12-core TRUS-guided biopsy were included into the study. From 52 patients, 519 targeted biopsies from 135 lesions and 1561 random biopsies were generated (total *n* = 2080). 8.9% (12/135), 29.6% (40/135), 40.7% (55/135), 11.9% (16/135), and 8.9% (12/135) lesions were classified as PiRADSv2 5,4,3, and 2 and unclassified, respectively.

The median target volume was 0.31 ml (IQR: 0.17–0.59) with median 3 (IQR: 2–5) biopsies per target, which corresponds to a biopsy density of 10.26/ml (IQR: 6.45–15.79). The median prostate volume was 49.3 ml (IQR: 37.82–73.32) (Table 1).

Overall detection of any PCa was 50.0 (26/52) for target and 59.6 (31/52) for random biopsy. The rate of clinically significant PCa was 44.23% (23/52) and 50.0% (26/52) for target and random biopsy, respectively (Table 2).

The subgroup analysis for specific PI-RADS classes with respect to the detection rates for any or clinically significant PCa is shown in Table 3. Detection rate for PI-RADS score 4 and 5 lesions only for all tumors and significant tumors dropped from 50.0% (26/52) to 44.0% (21/52) and from 44.23% (23/52) to 40.0% (21/52), respectively (Table 3).

The detection rate on a lesion based scale was 29.60% (40/135) and 22.96% (31/135) for overall and clinically significant PCa, respectively. Detection rate for peripheral versus central lesions was significantly higher for any and significant cancer (39.0% (29/75) versus 22.0% (13/60); *p* = 0.0233 and 32.0% (24/75) versus 15.0% (9/60); *p* = 0.0342) (Table 4).

We could not detect a volume dependent bias affecting the detection rate. There was no statistical difference in tumor detection rate between higher and lower target volumes (30.88% (21/68) versus 28.36% (19/67), *p* = 0.9). Even the lower interquartile 25% with volumes 0.17–0.31 ml showed no decreased rate of 35.29%  (12/34/). In a logistic regression analysis, tumor volume (*p* = 0.192; OR = 1.005) and biopsy density (number of biopsies per ml tumor volume) (*p* = 0.029; OR = 1.001) did also not show statistically significant risk bias for detecting PCa.

The distribution of different Gleason scores from detected PCa according to target biopsy or random biopsy is shown in Table 5. A significantly higher rate of bilateral tumors (*p* = 0.002) was detected in random biopsy compared to target biopsy (mean: 70.97 (±0.46; 22/31) versus mean: 32.26 (±0.48; 10/31); Table 5).

Specific Gleason upgrading (biopsy versus radical prostatectomy), defined as an increase in either the primary or the secondary pattern, was detected in 23.06% (6/26) and 11.54% (3/26) for target and random biopsy (*p* = 0.28; Table 5). Target biopsy detected significantly higher rates of Gleason 8–10 tumors (*p* = 0.0017; Table 5). No statistical difference was detected between detection rates for Gleason grades 6 and 7 (Table 5).

Sampling efficiency as the median number of cores needed to detect clinically significant PCa was 9 for target only (IQR: 6–14.0) and 32 (IQR: 24–32) for random biopsy. The utility as the number of additionally detected clinically significant PCa cases by either strategy was 0% (0/52) and 3.85% (2/52) for target and random biopsy.

Adverse events were reported in two patients: temporary bleeding and a rectum perforation. No infectious complications were reported.

## 4. Discussion

Previous studies regarding the detection rate of MRI/TRUS fusion biopsy of the prostate were summarized in the four recent systematic reviews by Robertson et al. 2013 [16], Marks et al. 2013 [17], Valerio et al. 2015 [11] (23.6% (range: 4.8–52%) for standard biopsy and 33.3% (range: 13.2–50%) for target biopsy), and Gayet et al. 2016 [10]. All reviews report a moderate advantage for the MRI/TRUS fusion target biopsy in the detection rate of clinically significant prostate cancer with fewer numbers of biopsies necessary.

Valerio et al. report an overall cancer detection rate of clinically significant prostate cancer with Gleason grading greater than or equal to 4 to be 33.3% (range: 13.2–50%) and 23.6% (range: 4.8–52%) for MRI/TRUS target biopsy and 12-core TRUS-guided transrectal biopsy as standard test. No overall statistically significant difference was detected. However, results from individual studies were heterogeneous with higher detection rates for either approach. Especially in the setting of repeat biopsy in men with persistent PSA elevation and prior negative 12-core transrectal biopsy, the MRI/TRUS fusion biopsy has shown superior results in detecting clinically significant prostate cancer [10, 18] and has been recommended in the European guideline for prostate cancer [19].

The systematic review by Gayet et al. [10] focused on different technical platform to perform mpMRI/TRUS fusion biopsies and their performance in detecting clinically significant prostate cancer. Detection rates for significant prostate cancer ranged between 35.7–43.4% and 28.5–36.8% for targeted and systematic random biopsy [10].

However, several other studies report no clear advantage of mpMRI/TRUS-guided targeted biopsy and recommend the combination of targeted and systematic biopsies for the detection of prostate cancer [12].

In this study, we report a series of 52 patients with suspected prostate cancer in a repeat biopsy situation with MRI/TRUS fusion target and random biopsy of the prostate using the novel MonaLisa system (Biobot surgical). All patients had received a previously negative 12-core transrectal TRUS-guided biopsy.

Almost all reported studies were tested against the standard 12-core transrectal biopsy. The strongpoint of our study clearly is the stringent use of a systematic volume adapted perineal random biopsy with high sampling density as control. Only by applying the same robot-guided perineal technique in either target or random mode, the true value of the MRI guided target biopsy can be evaluated.

In our series, we report a high detection rate in both biopsy modes comparable to the literature [10, 11, 16, 17]. Even small volumes were targeted correctly. A moderate advantage could be detected for cancer detection rate in random biopsy with 44.23% (23/52) and 50.0% (26/52) for target and random biopsy, respectively. Some of the reported studies in the reviews above have also reported higher detection rates for random biopsies [11]. The number of bilateral tumors was also approximately twice as high in random compared to target biopsy. We also detected a higher rate of Gleason Grade upgrading in target biopsy. However, approximately 4 times the number of biopsies were needed (32 (random) versus 9 (target)) to reach the same or improved detection rate.

The lesion based analysis showed low overall detection rates of 31.1% and 24.4% for any and significant cancer in our dataset. The latest (153 patients and 287 lesions) series published by Kesch et al. 2016 [20] using the same technique as we did shows similar detection rate of 34.8% and 29.9% for any and significant cancer, respectively. Another series (62 patients and 116 lesions) by Mertan et al. 2016 [21] and Cash et al. 2016 [22] (408 patients) reported similar results as well. The latest data including ours underline the poor performance of the PI-RADS scoring systems on a lesion based analysis.

The number of adverse events was acceptable. The reported rectum perforation occurred in a patient with the highest prostate volume of 141.9 ml of the series. It occurred early when adjusting the TRUS probe inside the rectum and did not happen due to high sampling number. The patient received immediate endorectal clipping of the lesion and fully recovered without further complications. Few case reports exist on this complications [23]. It might be underestimated complications because late onset of the symptoms might occur and most of the complications regarding transrectal ultrasound and/or biopsy of the prostate are reported within 24 h [6]. Therefore, high volume prostates will have to be treated with special caution, even though higher numbers of such patients will have to be analyzed in the future.

The value of the MRI/TRUS target biopsy using the technique described in our series provides heterogeneous results. On the one hand, we do not see an advantage in the detection rate of clinically significant tumors and understage tumor volume. On the other hand, we needed 4 times the number of biopsies to reach the detection rate with random biopsies. Economic implications are generated for target as well as for random biopsy. For target biopsy, a mpMRI and subsequent evaluation by a radiologist is needed for each patient. The low number of biopsies needed is clearly in favor of the workload by the pathologist for evaluating the biopsies histopathologically. On the other hand, random biopsy could spare the mpMRI and possible bias from the software based fusion process to the TRUS image. The workload for the pathologist, however, would be 4 times higher. The most important implication for a patient with suspected prostate cancer but previous negative 12-core transrectal biopsy is a high level of security regarding the status of the prostate. High numbers of biopsies do not present an increased infectious risk in the transperineal approach. General anesthesia is required in the transperineal setting to allow for precise planning and execution of the biopsies and optimal pain management.

## 5. Conclusions

The important strongpoint of our approach is the transperineal approach itself with excellent access to all areas of the prostate and the easy and quick planning of the biopsy positions, which allows optimal coverage of the high risk areas especially the apex and the anterior zone [13]. The most precise characterization of the tumor in our series is provided by the combination of the two methods. Our data suggests that there is potential to improve the radiological PI-RADS version 2 classification scheme in order to avoid future false positive findings and further reduce the number of false negatives. We hope that combination of target and random biopsies will enable us in the future to develop feedback correlations from target and random biopsies to the original MRI data to improve radiological classification.

## Figures and Tables

**Table 1:** Patient characteristics at MonaLisa biopsy (*n* = 52 patients, *n* = 135 MRI lesions).

	Mean/±SD/median/IQR
Age (years)	65.8/7.3/66.0/60.0–71.8
PSA (ng/ml)	9.9/5.9/8.8/5.7–13.04
MRI prostate volume (ml)	57.6/26.6/49.3/37.8–73.3
Number of lesions/patient (*n*)	2.6/1.5/2.0/1.0–4.0
MRI lesion volume (ml)	0.6/0.7/0.3/0.2–0.6
Number of biopsies (total)/patient (*n*)	39.8/40.0/36.3–43.0
Number of target biopsies/patient	10.2/4.8/9.0/6.0–14.0
Target biopsy density (*n*/ml lesion volume)	12.4/8.9/10.3/6.5–15.8
Number of random biopsies/patient	30.0/5.6/32.0/24.0–32.0

PSA: prostate specific antigen; MRI: magnetic resonance imaging.

**Table 2:** Detection rate of prostate cancer of target versus random biopsy.

	Any cancer^*∗*^ % (*n*/*n*)	Significant cancer^*∗∗*^ % (*n*/*n*)
Overall	59.6 (31/52)	51.9 (27/52)
Target biopsy	50.0 (26/52)	44.2 (23/52)
Random biopsy	59.6 (31/52)	50.0 (26/52)

*∗*: prostate cancer with any Gleason grade; *∗∗*: prostate cancer with Gleason grade ≥ 4.

**Table 3:** MRI lesion-based detection rate of target biopsies (*n* = 135).

	Any cancer^*∗*^ % (*n*/*n*)	Significant cancer^*∗∗*^ % (*n*/*n*)
PiRADS score		
5	83.0 (10/12)	75.0 (9/12)
4	45.0 (18/40)	35.0 (14/40)
3	18.0 (10/55)	13.0 (7/55)
2	6.0 (1/16)	6.0 (1/16)
Unclassified	25.0 (3/12)	17.0 (2/12)

PiRADS: Prostate imaging Reporting and Detection System.

*∗*: prostate cancer with any Gleason grade; *∗∗*: prostate cancer with Gleason grade ≥ 4.

**Table 4:** Detection rate of prostate cancer in peripheral versus central target biopsy.

	Any cancer^*∗*^			Significant cancer^*∗∗*^		
	Peripheral% (*n*/*n*)	Central% (*n*/*n*)	*p* value	Peripheral% (*n*/*n*)	Central% (*n*/*n*)	*p* value
Overall	39.0 (29/75)	22.0 (13/60)	0.0223	32.0 (24/75)	15.0 (9/60)	0.0342
PiRADS score						
5	75.0 (6/8)	100.0 (4/4)	0.32	75.0 (6/8)	75.0 (3/4)	0.99
4	56.0 (14/25)	25.0 (4/16)	0.052	44.0 (11/25)	19.0 (3/16)	0.10
3	27.0 (6/22)	12.0 (4/34)	0.14	23.0 (5/22)	6.0 (2/34)	0.065
2	9.0 (1/1)	0.0 (0/5)	—	9.0 (1/1)	0.0 (0/5)	—
Unclassified	22.0 (2/9)	100.0 (1/1)	—	11.0 (1/9)	100.0 (1/1)	—

PiRADS: Prostate imaging Reporting and Detection System.

*∗*: prostate cancer with any Gleason grade; *∗∗*: prostate cancer with Gleason grade ≥ 4.

**Table 5:** Tumor characteristics.

	Target biopsy only	Random biopsy	*p* value
Gleason score% (*n*/*n*)			
6	12.0 (3/26)	16.0 (5/31)	0.63
7	15.0 (4/26)	32.0 (10/31)	0.15
8–10	73.0 (19/26)	52.0 (16/31)	0.0017
Upgrading^*∗*^% (*n*/*n*)	23.1 (6/26)	11.5 (3/26)	0.28
Bilateral tumor			
Mean (±SD; *n*/*n*)	32.26 (0.48; 10/31)	70.97 (0.46; 22/31)	0.002

*∗*: specific Gleason upgrading (biopsy versus radical prostatectomy), defined as an increase in either the primary or the secondary pattern.
